# Urgent action needed: addressing the regulatory gap in e-cigarette trade and usage

**DOI:** 10.1057/s41271-024-00505-1

**Published:** 2024-07-05

**Authors:** Juan S. Izquierdo-Condoy, Esteban Ortiz-Prado

**Affiliations:** One Health Research Group, Faculty of Health Science, Calle de los Colimes y Avenida de los Granados, Universidad de Las Américas, 170137 Quito, Ecuador

**Keywords:** E-cigarette, Regulation, Public health, Low- and middle-income countries

## Abstract

Tobacco use is associated with serious health problems. Global efforts, such as the World Health Organization’s Framework for Tobacco Control, have reduced tobacco use, but challenges remain. Initially perceived as aids for smoking cessation, e-cigarettes have gained popularity among young people and non-smokers. Government approaches to regulating e-cigarettes range from treating them like tobacco, requiring a prescription for their use to outright bans. Although touted as a valuable alternative, evidence suggests that increased e-cigarette use carries potential direct and indirect health risks, necessitating urgent regulatory measures on a global scale. Lack of defined and uniform regulations poses substantial public health risks, compounded by marketing targeting vulnerable groups. Immediate interventions, public awareness, and research are essential to effectively control the current e-cigarette epidemic.

Tobacco consumption has been associated with numerous adverse health outcomes, early onset of malignancies, cardiovascular dysfunctions, cerebrovascular events, and cancer due to the vast array of noxious constituents present in tobacco [1, 2]. Over the past two decades, nations pivoted their strategic focus in tobacco control toward risk mitigation and behavioral modification with a plethora of successful interventions [3]. However, such efforts aimed at reducing tobacco use have rarely focused on enhancing social support as part of nations’ social capital [4].

The World Health Organization’s Framework for Tobacco Control (FCTC), endorsed by 182 countries, signifies a global commitment to addressing smoking cessation [5], currently includes measures such as restricting sales to minors, strategically concealing tobacco-derived products in retail environments, implementing enhanced tax strategies to deter consumption, employing graphic health warnings, and rigorously restricting tobacco-related media promotions [6]. Recent empirical evidence from the fourth World Health Organization (WHO) shows a reduction in tobacco-use globally from 1.32 billion users in 2015 to 1.30 billion in 2021. The WHO anticipates further decline to 1.27 billion by the year 2025 [7]. Yet, the WHO and the Pan American Health Organization (PAHO) report data suggesting that by 2020, tobacco users will constitute 22.3% of the global populace [2, 5]. We argue that now a much more effective approach would leverage emerging information, technologies, and materials to enhance human potential, both physical and intellectual.

Amidst the backdrop of traditional tobacco control, electronic nicotine delivery systems (ENDS), colloquially known as e-cigarettes, made their debut in China in 2003. Experts and groups such as the Royal College of Physicians originally proposed and conceptualized these devices as a cessation tool for entrenched tobacco users [8, 9]. Subsequently, these devices praised as an innovative and promising approach to mitigate the harmful health consequences associated with use of and addiction to conventional tobacco products [10, 11]. Global uptake of e-cigarettes has expanded notably, especially among individuals previously uninterested in tobacco cessation [12]. E-cigarettes have disproportionately attracted youth due to their design, variety of flavors, and targeted, aggressive marketing strategies by industry [13]. Although these devices have assisted some older adults to avoid traditional tobacco, regulatory efforts have been challenged by their rapid market expansion [14]. Evidence of the effectiveness of e-cigarettes in promoting smoking cessation is limited, and observers have noted the widespread use of e-cigarettes across various age groups, with particular risk for children aged 13–15 years [15]. This risk is threefold: exposure to harmful substances found in e-cigarette “e-juices,” increased risk of initiating tobacco cigarette use, and potential for e-cigarette use to serve as a gateway to use of more potent substances, such as tetrahydrocannabinol [16–18]. Evidence to date, however, suggests that legislation on the prohibition of electronic cigarettes (e-cigarettes) has reduced likelihood of consuming e-cigarettes, tobacco cigarettes, and even alcohol [19].

Although initially proposed as a “healthy alternative” to tobacco, the global adoption of e-cigarettes has not yielded the risk-reducing environment. The primary public health strategy currently is to regulate these products with at least the same rigor as applied to tobacco cigarettes [20]. Thus, governments and public health advocates have applied the Nuffield intervention ladder to e-cigarettes in various ways, from endorsing them for tobacco cessation to implementing outright bans [21]. Treating e-cigarettes as tobacco products is now is a prevalent approach, primarily because they contain nicotine [22]. Ecuador and El Salvador selected minimum age restrictions and usage constraints in enclosed spaces. Even so, users are able to access these products relatively easily due to insufficient oversight of sales [23–25]. Venezuela, Brazil, Argentina, Mexico, Uruguay, and more recently Nicaragua, Panama, and Suriname have imposed complete bans on e-cigarettes. These bans extend to the manufacture, export, import, sale, and possession of the devices [26].

Countries outside of the Americas, such as South Africa and Ghana, have chosen a less restricted approach. These nations regulate e-cigarettes as medicinal products, subject to the same rigorous controls required for other medicines [27]. This would restrict e-cigarette use to individuals with a doctor’s prescription for smoking cessation while protecting the broader population. This strategy is likely to impose burdens on the state, such as raising costs for standards-compliant e-cigarettes, thus limiting access for users [23, 25].

Designers of more nuanced strategies aimed to regulate specific e-cigarette ingredients, particularly nicotine and flavorings. Some jurisdictions have imposed a maximum permissible nicotine concentration of 20 mg/ml for marketed devices or have banned the sale of any nicotine-containing devices [23]. Research indicates that wide availability of flavors is a primary motivator for e-cigarette use among youth. Consequently, policymakers have proposed banning flavored e-liquids [28].

Some nations treat e-cigarettes as distinct products governed by specialized legislation. Canada’s Tobacco Products and Vape Act regulates the manufacture, sale, labeling, and promotion of e-cigarettes. This legislation aims to prevent e-cigarette use among youth and non-tobacco users while providing an alternative cessation tool for smokers [23, 29].

Proposals to reduce the harm of e-cigarettes to date have been based on the selective use of evidence. It is difficult to determine the exact role of these devices in the fight against tobacco smoking; even low levels of tobacco consumption are harmful to health. As the supply and, consequently, the demand for e-cigarettes continues to grow, the specific uses of e-cigarettes have not yet been fully explored and described so far, especially in lower resource nations [30].

Thus, the need to address the increasing prevalence of e-cigarette use is urgent, especially in less developed countries where misconceptions about their safety are rife. Among youth, high school students and those in grades 6–12 are increasingly hooked, mistakenly believing these devices are benign [31, 32]. In high resource countries, absence of strict regulations allows industry to strategically market e-cigarettes, and to target vulnerable groups such as adolescents. The situation is worse in low resource countries, where inadequate healthcare systems and less stringent regulations exacerbate the risks. Uncontrolled distribution and marketing of these poorly regulated and addictive products creates risk for public health disasters.

Lack of comprehensive policies leads to unchecked marketing tactics, unfettered use of the products in public spaces, and inconsistent quality assurance. These factors endanger individual and public health and impede scientific exploration into the health consequences of e-cigarettes. Without clear reporting standards and consistent manufacturing norms, researchers will find it to be exceedingly difficult to conduct extensive, long-term epidemiological research. Thus, healthcare professionals lack evidence to provide authoritative advice to patients on e-cigarette consumption.

Countries around the world with limited resources, including in Latin America, face substantial challenges. Socio-political and economic barriers often hinder the establishment and enforcement of effective public health policies. And sound policy requires recognition and distinguishing between target groups: those who need to reduce their risk by switching from tobacco to nicotine substitutes, and youth who view e-cigarettes as a status symbol or sign of maturity [20]. Governments need to develop regulatory measures tailored to the specific challenges and market dynamics of e-cigarettes in their jurisdictions.

The global community must unite to prioritize the establishment of comprehensive regulatory frameworks for e-cigarette legislation based on better information about the effects of these devices on public health. The health of present and future generations hinges on collective and timely actions to prevent a repetition of the history with tobacco cigarettes. Immediate interventions, public awareness campaigns, and targeted research efforts are crucial for limiting the e-cigarette epidemic.
